# Is 4‐month‐old infants' night waking affected by mothers' responses to them? A cross‐sectional survey in Japan

**DOI:** 10.1002/nop2.695

**Published:** 2020-11-27

**Authors:** Megumi Hazumi, Shun Nakajima, Yoshiko Adachi

**Affiliations:** ^1^ Department of Mental Health Policy and Evaluation National Center of Neurology and Psychiatry National Institute of Mental Health Tokyo Japan; ^2^ National Center for Cognitive Behavior Therapy and research Tokyo Japan; ^3^ Institute of Behavioral Health Fukuoka Japan

**Keywords:** infant, mother's response, night waking

## Abstract

**Objective:**

To investigate the association between night waking frequency in 3‐ to 4‐month‐old infants and mothers' response to them.

**Design:**

Cross‐sectional survey.

**Sample:**

We examined 663 mothers of infants aged 3–5 months who attended regular health checks for 4 months at 7 public health centres in Japan between September 2006 and March 2007.

**Measurements:**

Mother‐reported questionnaires were used, measuring the frequency of infants' night waking and four types of responses by mothers. Using multiple regression, the association between number of wakings and each response was evaluated adjusting for covariates, that is mother's (e.g. feelings of worry and bed‐sharing) and infant's (e.g. age and sex) demographic variables.

**Results:**

The number of wakings was related to “immediately feeding and/or checking diapers” (*β* = 0.16, *p* = .002).This response to infants' night waking may be associated with night waking frequency.

**Conclusion:**

Modifying caregiver responses to infants' night waking by reducing immediate feeding or diaper checks could improve infants' night waking frequency.

## 1 BACKGROUND

Sleep cycle develops gradually from the moment of birth, and 47%–81% of 4‐month‐old infants sleep without waking in the night (Henderson et al., 2011). Although frequent night waking does not seem to induce subsequent abnormal growth for under 6‐month‐old infants (Douglas & Hill, 2013), caregivers tend to consider it as the most serious sleep problem (Sadeh et al., 2011b). In fact, frequent night waking induces mental health problems in parents, such as parental stress, maternal depression and reduced sense of competence (Sadeh et al., 2011a; Teti & Crosby, 2012). Additionally, sleep disturbance—often induced by nocturnal waking in infants (Bayer et al., 2007)—is one of the factors related to physical health problems in infants (Nishihara et al., 2000). Therefore, it is important for mothers to control nocturnal waking in infants.

As well as biological factors (Henderson et al., 2011; Jian & Teti, 2016), environmental factors often affect nocturnal waking in infants. These factors include the nuclear family structure, living in a multiple‐dwelling house, being a young mother, being a working mother, breastfeeding and bed‐sharing (Anuntaseree et al., 2008; Fujiwara et al., 2016; Teti et al., 2016). Specifically, previous studies have demonstrated that caregiver behaviours as a response to night waking, such as immediate responses or stimulating behaviours that promote wakefulness, can affect night waking in over 6‐month‐old infants; further, night‐time interaction between the caregiver and infant is important for attachment development (Higley & Dozier, 2009). A cross‐sectional study of infants up to 36 months old indicated a relationship between night waking frequency and feeding for re‐sleep regardless of age (Mindell et al., 2010). A randomized controlled study indicated that avoiding behaviours such as immediate responding, turning the lights on and picking infants up decreases the number of night waking in 6‐ to 16‐month‐old infants (Gradisar et al., 2016). Further, a longitudinal study showed associations between making self‐soothing conditions and lower frequency of night waking in 12‐month‐old infants (Burnham et al., 2002). Thus, some kind of caregiver responses to night waking apparently promote night waking in older infants. However, the evidence is inadequate to infer that the same applies to younger infants, given the differences in sleep development between infants under 6 and over 6 months of age (Douglas & Hill, 2013).

Therefore, the relationship between caregiver response and night waking in younger infants remains unclear. Although an association between some response pattern to night waking and the presence of sleep problems in 4‐month‐old infants has been demonstrated (Hayama et al., 2008; Paul et al., 2016), the frequency of waking per night has not been previously accounted for. Further, although the environments such as bed‐sharing (Fukumizu, 2005) and responses of caregivers during daytime relate to the frequency of night waking in 3‐month‐old infants (Anuntaseree et al., 2008; Paul et al., 2016), caregiver responses, specifically when night waking occurs, have not yet been examined. Considering the findings of a previous study indicating the effect of unnecessary night‐time interventions in 4‐month‐old infants on subsequent slow progress of sleep regulation development up to 12 months old (Voltaire & Teti, 2018), it is possible that night waking may also be affected by night‐time intervention.

To the best of our knowledge, there is no evidence to determine whether the association between caregiver responses and night waking in infants over the age of 6 months applies to infants under 6 months old. Therefore, we aimed to investigate whether caregiver response is associated with night waking in younger infants by using a cross‐sectional survey based on mother‐reported questionnaires about infant sleep, health status, and feeding behaviour.

## 2 METHODS

This study employed a cross‐sectional design. We used the data collected at one city at the previous study and additional data collected for re‐examination the result of the previous study at various cities (Hayama et al., 2008).

### 2.1 Participants and procedure

Participants were mothers attending health checks for 4‐month‐old infants at seven public health centres in four prefectures (Kagoshima, Fukuoka, Okinawa, and Hokkaido) in Japan between September 2006 and March 2007. At the health check, infants and their parents are given a health examination by health professionals to ensure the healthy development of infants. All 4‐month‐old infants and their parents are invited to attend the health check in compliance with the law in Japan. The participation rate during the study was 93.4% (Ministry of Health, Labour and Welfare 2008).

Inclusion criterion was being a mother of an infant aged 3–5 months. Data collected from caregivers other than mothers were excluded to reduce the confounding effects of sex, age and family status of the respondents. Participants whose age of infants were unclear were also excluded because of difficulty in adjusting age.

### 2.2 Measures

The questionnaire used in this study measured a set of variables. The questionnaire was developed for the previous study (Hayama et al., 2008). Each item was developed based on several studies (Anuntaseree et al., 2008; Fujiwara et al., 2016; Fukumizu, 2005; Jian & Teti, 2016; Mindell et al., 2010), which was designed to be concise so that even mothers with infants could answer the questions without any burden in order to obtain a high response rate.

First, the frequency of waking at night, which served as the outcome measure, was assessed. We defined night‐time as the hours between 0 a.m. and 6 a.m. The average number of infant waking per night was subjectively reported by the mothers. They were asked "How many times does your infant awake at night?" Multiple responses were rounded to the median (e.g. “2 or 3 times” was computed as “2.5 times”), and the measure was treated as a continuous variable.

Second, the participants were asked "How do you respond when your baby cries at night while you are sleeping (choose any number of options)?" to indicate their usual responses to infant waking from among four options: “immediately feed and/or check diapers,” “hold the baby and soothe him/her,” “check the baby's bedding and/or clothes” and “do not respond immediately but wait and watch for a while.” These were treated as binary variables (1: chosen/0: not chosen). These items were generated based on previous studies identifying recommended caregiver responses to night waking in infants (Mindell et al., 2010).

Third, for demographic information, response to a series of items were recorded: mother's age; delivery experience (1: primipara/0: multipara); employment (1: employed/2: unemployed); feelings of worry (1: yes/0: no); breastfeeding (1: yes/0: no); residential type (1: multiple‐dwelling house/0: single‐dwelling house); family structure (1: multiple generations living together/0: nuclear family); and presence of bed‐sharing (1: yes/ 0: no). These variables were considered to be covariates based on the findings of prior studies. For example, the relationship of age and sex with sleep in 3‐ to 6‐month‐old infants was previously demonstrated (Jian & Teti, 2016). Factors such as the nuclear family structure, living in a multiple‐dwelling house, being a young mother and being a working mother have been reported as risk factors for some kind of caregiver response (Fujiwara et al., 2016), while breastfeeding has been reported as a risk factor for infant nocturnal waking (Anuntaseree et al., 2008). Further, bed‐sharing was included because mothers who share beds with infants tend to report a higher frequency of night waking (Fukumizu, 2005). Information for infants such as age, sex, weight, and time in bed were also reported.

### 2.3 Analysis

The average and standard deviation of all continuous variables and the frequency and proportion of all categorical variables were calculated to describe the sample characteristics in this study. A frequency distribution of infant waking was created.

After conducting a correlation analysis among the variables, a multiple linear regression analysis was performed to evaluate the association between the frequency of waking and the four caregiver response types, adjusting for mother's age; feelings of worry; bed‐sharing; breastfeeding; house information; family structure; and infant's age, sex, weight and time in bed. The analyses were performed after excluding the data for the participants who reported waking more than three times per night, based on a systematic review of previous literature identifying the cut‐off point of problematic sleep in 3‐ to 6‐month‐old infants as more than three times per night (Galland et al., 2012).

All analyses were performed using SPSS Statistics version 25 (IBM Japan, Chuo‐ku, Tokyo, Japan) with pairwise deletion for missing data. Statistical tests were two‐tailed, and the significance level was set at *p* < .05.

### 2.4 Ethical approval

All procedures were approved by the on Ethics Committee of the Association for Preventive Medicine of Japan (approval number: 18001).

## 3 RESULTS

### 3.1 Sample characteristics

**Figure 1:**
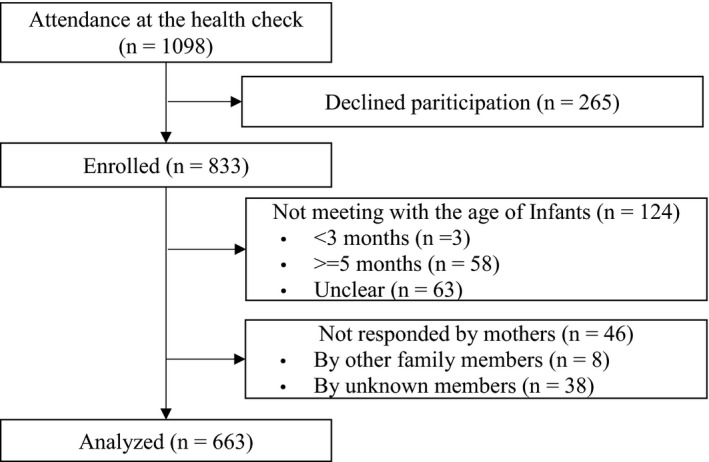
Flow chart illustrating participant recruitment and final case selection

Of the 1,098 parents who attended at the medical examination, the data of 663 were analysed as shown in Figure 1.

**Figure 2:**
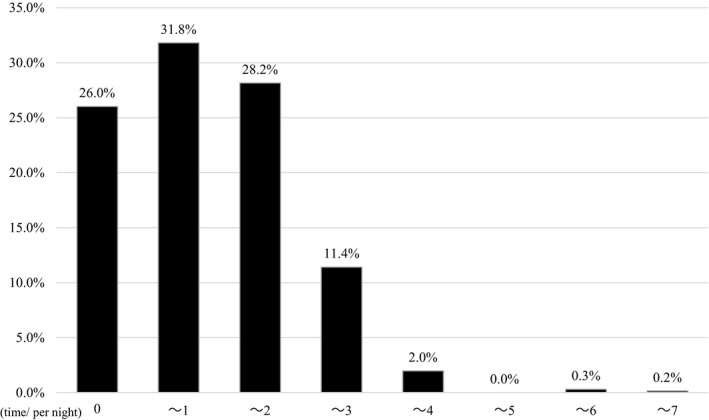
Distribution of nocturnal waking frequency in 4‐month‐old infants

The mean age of infants was 119.4 days and 48.0% were male (Table 1). Mean number of night wakings was 1.24 times; 26.0% were reported to sleep without waking until the next morning; whereas, 2.5% were reported as waking up over three times per night (Figure 2).

**Table 1:** Sample characteristics (*N* = 663)

	Mean or *N*	*SD* or %
Number of night waking in infants (times)	1.24	1.03
“Do not respond immediately but wait and watch for a while”	218	32.9%
“Immediately feed and/or check diapers”	508	76.6%
“Hold up the baby and soothe him/her”	160	24.1%
“Check the baby's bedding and/or clothes”	84	12.7%
Delivery experience (primipara yes = 1)	411	57.8%
Solely breastfeeding (yes = 1)	350	52.8%
Family structure (nuclear family = 1)	524	79.0%
Residential type (apartment = 1)	417	62.9%
Age of mother (years)	30.0	4.88
Employed (yes = 1)	73	10.7
Bed‐sharing (yes = 1)	383	57.8%
"Feelings of worry" (yes = 1)	36	5.4%
Age of infant (days)	119.4	13.36
Sex (male)	318	48.0%
Weight (g)	6,718.0	875.02
Total sleep time of infant (hr)	7.7	3.98

### 3.2 Variables related to the number of nocturnal waking

Table 2 shows the correlations between all the study variables. The number of waking was weakly correlated with “do not respond immediately but wait and watch for a while” (*r *= −0.15, *p* < .001), “immediately feed and/or check diapers” (*r* = 0.23, *p* < .001), multipara (*r *= −0.14, *p* < .001), solely breastfeeding (*r* = 0.17, *p* < .001) and bed‐sharing (*r* = 0.16, *p* < .001).

**Table 2:** Correlations between the variables

		1	2	3	4	5	6	7	8	9	10	11	12	13	14	15	16
1	Number of night wakings																
2	“Do not respond immediately but wait and watch for a while”	−0.15***															
3	“Immediately feed and/or check diapers”	0.23***	−0.53***														
4	“Hold up the baby and soothe him/her”	0.02	0.07	0.01													
5	“Check the baby's bedding and/or clothes”	0.04	0.04	0.04	0.16***												
6	Delivery experience (primipara or not)	−0.14***	0.05	−0.09*	0.10**	0.01											
7	Solely breastfeeding	0.17***	−0.09*	0.10**	−0.05	−0.05	−0.01										
8	Family structure	0.09*	0.03	0.05	0.05	0.10**	−0.03	0.07									
9	Residential type	−0.04	−0.06	0.09*	0.03	−0.01	0.14**	−0.00	−0.32**								
10	Age of mother	0.06	−0.01	0.00	0.02	−0.03	−0.32***	−0.04	−0.01	−0.13**							
11	Employment status	−0.01	−0.01	0.00	0.03	−0.01	0.14***	−0.14***	−0.09*	0.07*	−0.06						
12	Bed‐sharing (yes)	0.16***	0.11**	0.13**	0.05	0.04	0.12**	−0.12**	0.01	0.10**	0.01	0.03					
13	Feelings of worry	0.04	0.03	0.00	0.01	0.08*	0.04	0.00	0.09*	−0.01	−0.01	0.06	−0.01				
14	Age of infant	0.04	−0.08*	0.03	−0.02	0.03	−0.03	−0.01	−0.09*	0.04	0.09*	−0.01	−0.03	−0.03			
15	Sex	0.06	−0.02	0.03	0.05	−0.03	−0.07*	−0.02	−0.02	−0.07	0.09*	0.04	−0.05	−0.02	−0.00		
16	Weight	0.07	−0.04	0.03	0.11*	0.07	−0.12**	−0.00	0.05	0.00	0.03	0.01	−0.05	−0.03	0.32**	−0.36**	
17	Total sleep time	−0.06	0.01	−0.02	−0.10**	0.03	0.00	−0.02	−0.03	−0.10**	−0.05	−0.06	−0.05	−0.01	0.07*	−0.01	0.06

*<0.05,**<0.01,***<0.001

As seen in the result of the multiple linear regression analysis (Table 3), the number of waking was weakly related to “immediately feed and/or check diapers” (*β* = 0.17, *p* = .001) and was independent from solely breastfeeding (*β* = 0.15, *p* < .001), bed‐sharing (*β* = 0.14, *p* = .001) and multipara (*β* = −0.10, *p* = .04).

**Table 3:** Variables contributing to nocturnal waking in 4‐month‐old infants

	B	*SD*	*β*	95% CL	*p*
“Do not respond immediately but wait and watch for a while”	−0.07	0.09	−0.04	(−0.25 to 0.12)	.47
**“Immediately feed and/or check diapers”**	**0.35**	**0.11**	**0.17**	**(0.14** to **0.56)**	.001***
“Hold up the baby and soothe him/her”	0.06	0.09	0.03	(−0.12 to 0.24)	.52
“Check the baby's bedding and/or clothes”	0.05	0.11	0.02	(−0.18 to 0.27)	.67
**Delivery experience (primipara or not)**	**−0.17**	**0.08**	**−0.10**	**(−0.33** to **−0.01)**	**.04**
**Solely breastfeeding**	**0.26**	**0.08**	**0.15**	**(0.11** to **−0.41)**	.001***
Family structure	0.25	0.13	0.09	(−0.01 to 0.51)	.06
Residential type	−0.02	0.09	−0.01	(−0.18 to 0.15)	.85
Age of mother	0.00	0.01	0.01	(−0.01 to 0.02)	.84
Employment status	−0.02	0.12	−0.01	(−0.26 to 0.22)	.85
**Bed‐sharing (yes)**	**0.20**	**0.08**	**0.11**	**(0.05** to **0.35)**	**.01**
Feelings of worry	0.13	0.16	0.03	(−0.19 to 0.45)	.42
Age of infant	0.00	0.00	0.04	(0.00 to 0.01)	.38
Sex	−0.07	0.08	−0.04	(−0.23 to 0.09)	.40
Weight	0.00	0.00	0.01	(0.00 to 0.00)	.81
Total sleep time	−0.01	0.01	−0.07	(−0.03 to 0.00)	.12

Note

*R* ^2^ = 0.12***

Bold Values indicate *p*<.005.

*p*<0.001

The regression model had a mild to moderate fit to the data (*R* ^2^ = 0.12).

## 4 DISCUSSION

In this study, we aimed to examine the variables contributing to the number of night waking in 4‐month‐old infants using a cross‐sectional design and a self‐reported questionnaire. We found that 26% of the 4‐month‐old infants sustained sleep until the next morning and approximately 70% woke at least once per night. Furthermore, in the infants, the caregiver's common response to the infant's night waking was “immediately feeding and/or checking diapers,” which was positively and independently associated with the frequency of waking per night for breastfeeding, bed‐sharing and multipara, even after adjusting for other factors. Thus, responding immediately when 4‐month‐old infants wake at night may be a factor causing frequent night waking in younger infants.

Regression analysis showed that “immediately feeding and/or checking diapers,” breastfeeding, bed‐sharing and multipara associated with the frequent night waking. This indicated that immediate caregiver response to night waking may be one of the factors sustaining night waking frequency along with other biological factors, such as development of circadian rhythm stability (Anders, 1994; Goodlin‐Jones et al., 2001). Given that the response patterns other than “immediately feeding and/or checking diapers” in this study were not related with night waking, it is likely that night waking was affected by the speed of responses to infants, not by the type of responses to infants. The association between immediate response and frequent night waking may be accounted for by the operant conditioning theory that parental responses to night waking behaviours function as rewards for infants and may reinforce night waking, as 4‐month‐old infants' general behaviour patterns are modified by the parenting styles and a shorter time interval from behaviour to response tends to build stronger reinforcement(Commons et al., 1987; Sirvinskiene et al., 2012). On the other hand, it is known that ignoring infants' cues at night is likely to inhibits healthy attachment development (Higley & Dozier, 2009). Therefore, it is important for caregivers to take time to determine whether there is a real need to respond to the infant, rather than responding immediately or avoiding responding.

Consistent with previous studies, solely breastfeeding had a significant but weak association with frequent night waking. The same relationship was reported in several studies for infants aged under 6 months (Anuntaseree et al., 2008; Bayer et al., 2007; Galland et al., 2012). This suggests that the relationship between feeding and night waking may be common across cultures or countries. Indeed, a systematic review indicated that inappropriate feeding could induce frequent night waking (Douglas & Hill, 2013). Furthermore, breastfeeding is said to be difficult when performing proper feeding while the mother has low breast milk supply and experiencing physical discomfort (Ong et al., 2014). These uncomfortable experiences at breastfeeding might stimulate arousal revel in infants.

A weak but significant relationship was found between frequent night waking and bed‐sharing, which indicates that bed‐sharing promotes night waking. Bed‐sharing is known to increase various health risks such as sudden infant death syndrome and other sleep‐related infant deaths (Moon & Task Force on Sudden Infant Death Syndrome, 2016). There is some possibility that frequent night waking induced by bed‐sharing is signal symptom of such serious risks considering physical discomforts interfere sleep (Baron et al., 2019). Previous studies reported similar relationships in early infants (Fukumizu, 2005; Teti et al., 2016), although this relationship was not found in 3‐month‐old infants in Thailand (Anuntaseree et al., 2008). The discrepancies between studies might be due to the difference in age range of the infants as well as cultural difference.

Multipara mothers reported more frequent night waking than primipara mothers. Although the reason of the difference remains unclear, it is possible that the difference in amount of the interactions with infants in primipara and multipara affects the night waking. The amount of daytime interaction, which is said to affect the frequency of night waking (Paul et al., 2016), has been shown to be less among multipara than primipara mothers (Kalomiris & Kiel, 2016). These relationships might be mediated by the tendency to have fewer responses during daytime from primipara mothers.

The distribution representing the frequency of waking per night in 4‐month‐old infants suggests that 26% sustain sleep until the next morning and infants waking under three times per night were 71.4%. The findings of this study are consistent with a systematic review indicating that most 3‐ to 6‐month‐old infants wake less than three times per night (Galland et al., 2012).

Some limitations of the present study must be noted. First, sampling biases may be present, as the data were collected 13 years ago for the original study and additional study. Data collection was restricted to the western region of Japan and difficult to conduct with strict design under administrative environment. For this reason, a priori sample size calculation could not be conducted for the study. These biases may have influenced the proportion or the average values in the descriptive results owing to the differences in the availability of resources such as quality of health service or amount of accessible information among different regions. Nevertheless, the data was valuable because there are few studies conducted with government health service in Japan. Another bias may occur due to the increase in mothers' age at childbirth and decline in birth rates in the past 10 years. However, the differences in proportions or average values do not interfere with the results of the regression analysis, which, in our study, indicate non‐significant associations between infants' nocturnal waking and mother's age. Therefore, the relationship between immediate response and frequency of night waking may also be applicable to infants in other areas or in different generations. Second, since the questionnaire responses were subjectively reported by the mothers, there is the possibility of an overestimation in reporting as compared to the objective events, as suggested by a previous study indicating this disparity (Sadeh, 1996). However, we made an effort to minimize this bias by adjusting for factors that distort subjective evaluation by including these possibilities in the analysis, such as bed‐sharing and worrying. Third, the study does not offer strong evidence for causal associations because of its cross‐sectional design. Also, considering *R* ^2^ = 0.12, the regression model in this study could not explain all of the relationships with night waking. A longitudinal observational study and the study to explore potential factors could be conducted to lend support to this initial evidence base.

## 5 CONCLUSION

In conclusion, the current cross‐sectional survey with mothers of 3‐ to 4‐month‐old infants found a significant relationship between frequent night waking and immediate care response to night waking. This finding indicates that mothers of young infants could benefit from making sure their infants' safe carefully and calmly responding instead of providing an immediate response automatically.

## CONFLICT OF INTEREST

There are none to declare.

## AUTHOR CONTRIBUTIONS

M H contributed to data analyses, drafting the manuscript, and editing the manuscript. S N contributed to data analyses and editing manuscript. Y A contributed to study conceptualization, organization of the cohort, data collection, data analyses and reviewing and editing the manuscript.

## Data Availability

Not available.
